# Histone acetylation: a key determinant of acquired cisplatin resistance in cancer

**DOI:** 10.1186/s13148-023-01615-5

**Published:** 2024-01-03

**Authors:** Abhiram Natu, Tripti Verma, Bharat Khade, Rahul Thorat, Poonam Gera, Sangita Dhara, Sanjay Gupta

**Affiliations:** 1 Epigenetics and Chromatin Biology Group, Gupta Lab, Cancer Research Institute, Advanced Centre for Treatment, Research and Education in Cancer, Tata Memorial Centre, Kharghar, Navi Mumbai, MH 410210 India; 2 Homi Bhabha National Institute, Training School Complex, Anushakti Nagar, Mumbai, MH 400094 India; 3 Laboratory Animal Facility, Advanced Centre for Treatment, Research and Education in Cancer, Tata Memorial Centre, Kharghar, Navi Mumbai, 410210 India; 4 Biorepository, Advanced Centre for Treatment, Research and Education in Cancer, Tata Memorial Centre, Navi Mumbai, 410210 India; 5 Fuel Chemistry Division, Bhabha Atomic Research Centre, Trombay, Mumbai, 400085 India

**Keywords:** Cisplatin, Resistance, Hyperacetylation, PI3K, 2DG

## Abstract

Cisplatin is an alkylating class of chemotherapeutic drugs used to treat cancer patients. However, cisplatin fails in long-term treatment, and drug resistance is the primary reason for tumor recurrence. Hence, understanding the mechanism of acquirement of chemoresistance is essential for developing novel combination therapeutic approaches. In this study, in vitro cisplatin-resistant cancer cell line models were developed. Gene ontology and GSEA of differentially expressed genes between parental and resistant cells suggest that PI3K-AKT signaling, central carbon metabolism, and epigenetic-associated phenomenon alter in cisplatin-resistant cells. Further, the data showed that increased glucose transport, alteration in the activity of histone-modifying enzymes, and acetyl-CoA levels in resistant cells paralleled an increase in global histone acetylation. Enrichment of histone acetylation on effectors of PI3K-AKT and glycolysis pathway provides evidence of epigenetic regulation of the key molecules in drug resistance. Moreover, cisplatin treatment to resistant cells showed no significant changes in histone acetylation marks since drug treatment alters cell epigenome. In continuation, targeting PI3K-AKT signaling and glycolysis leads to alteration in histone acetylation levels and re-sensitization of resistant cells to chemo-drug. The data provide evidence of histone acetylation's importance in regulating pathways and cisplatin-resistant cells' cell survival. Our study paves the way for new approaches for developing personalized therapies in affecting metabolic pathways and epigenetic changes to achieve better outcomes for targeting drug-resistant cells.

**Supplementary Information:**

The online version contains supplementary material available at 10.1186/s13148-023-01615-5.

## Introduction

Cisplatin, a first-generation platinum drug, is used as a first-line therapy in clinical practice for treating various solid tumors, including ovarian, cervical, lung, stomach, head and neck, liver, and breast cancers [1]. Its efficacy has been demonstrated in previous studies, attributed to its ability to produce a variety of DNA-platinum adducts, resulting in double-stranded DNA damage and triggering mitochondrial apoptosis [2]. Despite being effective against a variety of cancer types, cisplatin treatment frequently encounters resistance, which results in treatment failure.

The mechanism of cisplatin resistance involves altered signal regulation networks in tumor cells, a strong ability to prevent apoptosis, decreased drug absorption or greater drug effusion and DNA damage repair capacity. The potential attenuation of DNA damage-mediated apoptotic signals, TP53 inactivation and the MAPK pathway gives tumor cells to develop cisplatin resistance. Moreover, PI3K/AKT, NF-κB, Stat3, and other signaling pathways are also implicated in the control of cisplatin resistance. Furthermore, recent research has demonstrated that cisplatin-resistant cancer cells showed alteration in metabolism, including biosynthesis, energy substrates, redox homeostasis, and signal transmission.

Drug-resistant cells' altered epigenetic and genetic makeup plays a crucial role in deregulating signal transduction mechanisms and metabolic reprogramming to meet energy and growth needs required for the survival of cells. Significant pathways, such as PI3K/Akt and tyrosine-kinase-based pathways, play a role in glucose metabolism and lipid synthesis for membrane formation. Inhibitors of these signaling pathways are currently being tested as part of combination treatments [3, 4]. Moreover, the interplay between cancer metabolism and substrates for epigenetic modifications is crucial in regulating the activity of epigenetic modifiers and gene expression [5].

Reprogramming of epigenetic modifications have demonstrated their prognostic value and linked to tumor aggressiveness, survival rates, metastasis and therapeutic resistance regardless of the cancer's origin. The changes in histone-modifying enzyme expression have been linked to cisplatin resistance in cancers such as non-small cell lung cancer [6, 7]. Moreover, overexpression of the long non-coding RNA TUG1 leads to epigenetic silencing of the PDCD4 gene, which affects cisplatin resistance in esophageal squamous cell carcinoma cells [8]. In breast cancer cells, miR-381 downregulates the drug transporter protein ABCB1/MDR1, which influences cisplatin sensitivity [9].

Despite the widespread use of cisplatin, only 19–33.8% of patients show a response to cisplatin in these cervical and liver cancer [10, 11]. Therefore, in-depth understanding the dysregulation and intricate cross-talk between epigenetic and metabolic reprogramming in cisplatin resistance of cervical or liver cancer cells will potentially provide a molecular basis for personalized cancer therapy in tumor resistance. The data revealed that cisplatin-resistant cervical and liver cancer cells have shown a higher abundance of histone acetylation marks on the promoter of genes related to PI3K subunits and glycolysis. Moreover, the chromatin state in cisplatin resistance is regulated by the PI3K signaling pathway indicated by inhibitor-based experiment. Finally, inhibiting glycolysis using 2-DG in cisplatin-resistant cells was found to decreases tumor growth. These findings suggest that the defining epigenetics and targeting metabolic processes as a promising new approach in cisplatin-resistant phenotype.

## Materials and methods

### Cell culture and chemicals

HeLa and HepG2 cell lines were procured from ATCC and further confirmed with STR profiling. Parental and cisplatin-resistant cell lines HeLa & HepG2 were cultured in DMEM (GIBCO), supplemented with heat-inactivated 10% fetal bovine serum (GIBCO). Cell lines were cultured at 37 °C in 5% CO_2_ incubator with humidified conditions. The cell culture was carried out in TPP tissue culture grade platicwares. Cisplatin (Pfizer), Wortmamin(CST) and 2DG(Sigma) were procured for the study. The cisplatin resistant cells for HeLa and HepG2 were developed as discussed previously [12]. The Resistance index (RI) was calculated by formula RI = IC_50_ of resistant cells/IC_50_ of parental cells.

### Cell viability assay

Equal number of parental or resistant cells (~ 500 to 1000 cells/well) were treated with increasing concentration of cisplatin for 24 h in 96 well plate, followed by growing in drug-free media for 48 h. 20 μl of MTT reagent (5 mg/ml) was added onto cells, and plate was incubated for 3 h. Crystals were solubilized using 100 µl of (10% SDS, 1% isopropanol, 0.1% HCL) buffer. Absorbance was measured at 540 nm in Biotek plate reader to calculate percent cell viability.

### Clonogenic assay

Cells were treated with the drug for 24 h and allowed to grow for 48 h in drug-free media. Then, 1000 cells from all experimental sets were plated, and allowed to grow for ~ 14 days. Cells were fixed with 4% paraformaldehyde for 15 min, and washed with 1X PBS thrice. Cells were stained with 0.5% crystal violet for 20 min and washed with distilled water five times. Colonies were counted manually.

### Cisplatin uptake assay

Cisplatin uptake was estimated using Total Reflection X-ray Fluorescence (TXRF). TXRF is an advanced variant of EDXRF used for trace and ultra trace elemental analysis in variety of matrices [13]. ~ 10^6^ cells were treated with 20 µM of cisplatin for 24 h. Cells were harvested dissolved in concentrated nitric acid in a water-bath for 6–8 h at 70 °C. Cisplatin standards were processed to calculate the exact concentration of platinum. The clear supernatant was taken for platinum measurement using TXRF.

### RNA isolation and cDNA synthesis

RNA extraction from cell lines was carried out by TRIzol method (Invitrogen #5596026) as per manufacturer's instructions. RNA concentration and quality were estimated based on absorbance at 230 nm, 260 nm, and 280 nm using Nanodrop (NanoDrop 2000c Spectrophotometer). Then, 2 µg of RNA was further treated with DNase I (Fermentas, #ENO521) for 30 min at 37 °C to remove any DNA contaminant. DNase I enzyme was inactivated by adding 2 µl of 25 mM EDTA followed by incubating samples at 65 °C for 10 min. DNase I treated-RNA samples (1000 ng) were subjected to cDNA synthesis using Thermoscientific Revert-Aid cDNA synthesis kit (Fermentas, #K1632). cDNA synthesis was carried out using random hexamer primers.

### RNA sequencing and data analysis

Samples with RIN value > 7 were selected for library preparation. A modified NEBNext RNA Ultra II directional protocol was used to prepare the libraries for total RNA sequencing. Sequencing was carried out on Illumina HiseqX to generate reads. Sequencing was done in biological duplicates. Reads were aligned to reference transcriptome using salmon tool to obtain counts. Further, counts were subjected to DESeq2 library in R for estimating differential gene expression. Gene ontology and pathway analysis was carried out using GSEA software (v4.1) or DAVID tool. Network analysis was carried out in cytoscape v-3.8 in continuation with GSEA.

### qPCR

cDNA template (5 ng) was used to setup qPCR reaction with SYBR™ Green dye master mixer (Applied Biosystem, #4367659). PCR was performed using ABI 7500 machine in 384-well plates. The amplification was carried out for 40 cycles. Cycle threshold (Ct) values obtained from the run were used for ΔΔCt method of data analysis. Primers used for qPCR are provided in Table 1 and 2.

**Table 1:** Primer sequences for qPCR

Gene	GeneID	Forward (5′–3′)	Reverse (5′–3′)
PIK3CA	5290	TTGTCGTGCATGTGGGATGT	CTGGTCGCCTCATTTGCTCA
PIK3CB	5291	GTAGACTGGGCCTTTGGAACTT	TGATGAGGCTGGGAAACAAGG
PIK3R1	5295	CCTGGCCTGAGAAGGTTTGG	GTCCCTTGTGAGTGGACGTG
HK2	3099	TTGACCAGGAGATTGACATGGG	CAACCGCATCAGGACCTCA
ENO1	2023	GTACATCTCGCCTGACCAGC	CTGGCTGTGAACTTCTGCCA
XIAP	331	ACGGGCTAAGGTAGGGTAGAG	CTAATGCTGGGGCGGTAGTC
BCL-XL	598	TGCCACCAGGAGAACCACTA	TCTGAGGCCAAGGGAACTGA
HDAC1	3065	ATATCGTCTTGGCCATCCTG	TGAAGCAACCTAACCGATCC
HDAC2	3066	GGGAATACTTTCCTGGCACA	ACGGATTGTGTAGCCACCTC
HDAC3	8841	TGGCATTGACCCATAGCCTG	GCATATTGGTGGGGCTGACT
HDAC4	9759	TCGCTACTGGTACGGGAAAAC	AGAGGGAAGTCATCTTTGGCG
HDAC5	10014	ACTGTTCTCAGATGCCCAGC	TGGTGAAGAGGTGCTTGACG
HDAC6	10013	AGTGGCCGCATTATCCTTATCC	ATCTGCGATGGACTTGGATGG
HDAC7	51564	TTCCTGAGTGCAGGGGTAGT	CATCGCCAGGAGGTTGATGT
HDAC8	55869	ATAACCTTGCCAACACGGCT	CTTGGCGTGATTTCCAGCAC
HDAC9	9734	ACTGAAGCAACCAGGCAGTC	TTCACAGCCCCAACTTGTCC
HDAC10	83933	CTGGCCTTTGAGGGGCAAAT	CAGCAGCGTCTGTACTGTCA
HDAC11	79885	CCGGAAAATGGGGCAAAGTG	TAAGATAGCGCCTCGTGTGC
HAT1	8520	AATGGCGGGATTTGGTGCTA	TCATCCCCAAAGAGTTGATGGG
KAT2A	2648	TCGAGTTCCATGTCATCGGC	TCAAGGCCAGAGTCTTGTGC
KAT2B	8850	GAATCGCCGTGAAGAAAGCG	TTATTTGCTGCAGGTCGGCT
KAT5	10524	AGCGTGAAGGACATCAGTGG	AGTCCGTTCTTAGTGGGGGT
CREBBP	1387	TGTTTTCGCGAGCAGGTGA	GTGCTGTCATTCGCCGAGA
EP300	2033	GCAGTGTGCCAAACCAGATG	GGGTTTGCCGGGGTACAATA
KAT8	84148	TCGGAGAAACGTACCTGTGC	TCTTGTCTACCCACTCGTCCA
RPS13	6207	GCTCTCCTTTCGTTGCCTGA	ACTTCAACCAAGTGGGGACG

**Table 2:** Primer sequences for ChIP-qPCR

Promoter binding site for gene	Forward (5′–3′)	Reverse (5′–3′)
PIK3CA	TATCTCTACCCCAGCTCGCC	GGGCTGCTCTTCTGACGTTT
PIK3CB	GCGCCCTATCCTCACAAAGG	TGCTGGCCTTTTCTTCCCAG
PIK3R1	CAGGAATTGCAGGGCCTCTC	CTGGCTGCGTCTCTAATCCG
HK2	TGATTGCCTCGCATCTGCTT	TGATGGAGGCCAGACCACTT
ENO1	TTCGGCTCACCGGTCCTAT	GGCTGCAGTAGCGTGGAAA

### In vivo tumorigenic assay

All animal experiments were performed under Institutional Animal Ethics Committee– approved protocol and institutional guidelines for the same were followed (Proposal No 18/2017). Cell line xenograft mouse model was developed with parental/HeLa or CisR/HeLa cell line. For the study, 6–8 week old NOD-SCID mice were used. ~ 3 million cells were subcutaneously injected into the right hind flanks and allowed to form tumor. After tumor development, animals were randomized to achieve mean tumor volume of 100-150mm^3^ for experimental group. Then, cisplatin (2.5 mg/Kg) or 2-DG (500 mg/Kg) was administered intra-peritoneally, twice a week. Tumor growth was monitored once in week by measuring size of tumor using a caliper. Tumor volume was calculated as (0.5 × LP^2^) where L is the largest dimension and P is perpendicular dimension. The tumor tissues were snap frozen or fixed for paraffin block preparation and sectioned for H&E staining.

### Glucose uptake

Glucose uptake was monitored as per manufacturer's instructions for Glucose Colorimetric Detection Kit (EIAGLUC, thermos fisher scientific). The supernatant from growing cells were collected and diluted up to 1:40 in assay buffer. Diluted samples were incubated with HRP, glucose oxidase, and substrate. Reaction was incubated for 30 min and measured at 560 nm using Spectrostar Nano Biotek LabTech plate reader. Percent glucose uptake was calculated based on absorbance obtained for culture media control.

### HAT and HDAC assay

The enzyme activity for HDACs and HATs was measured using colorimetric activity assay kits from BioVision (BioVision Research Products, USA) catalog #K331 & #K332, respectively. The nuclei extract from equal number was processed for activity assay as per manufacturer instructions. The nuclear extract was incubated with a colorimetric substrate followed by the developer, producing a chromophore. The chromophore can be easily analyzed using a Spectrostar Nano Biotek LabTech 96-well plate reader.

### Estimation of acetyl-CoA

Acetyl-CoA levels were measured using Acetyl-Coenzyme A Assay Kit, (#MAK039, Sigma). Acetyl-CoA concentration was determined based on fluorometric (ex = 535/em = 587 nm) product by coupled enzyme assay proportional to the metabolite present. The samples were prepared from ~ 0.1 million cells for experimental set. Sample was deproteinization using 25% (wt/vol) TCA final concentration followed by neutralization using 5 M KOH solution till pH 7–8 was achieved. Further, samples were processed for acetyl-CoA measurement as per manufacturer's instructions.

### AnnexinV-FITC/PI staining

 ~ 0.1 million cells were treated with the desired drug for 24 h as per the experimental setup. Cells were resuspended in 100 µl of 1X annexin binding buffer. 2 µl of annexin-FITC was added in each tube and incubated for 15 min in dark at room temperature for staining. Then 400 µl 1X annexin binding buffer along with 5 µl PI was added in each tube. Cells were incubated at 37 °C for 5 min. Staining controls like unstained cells, only annexinV-FITC and only PI-stained cells were used for gating. Percent apoptotic and necrotic populations were calculated by Attnue NxT flow cytometry and analyzed using Cell Quest Software.

### Chromatin immunoprecipitation (ChIP)-qPCR

4 million cells per IP reaction were fixed using cross-linking reagent 1% formaldehyde for 10 min, and then the reaction was quenched using 125 mM glycine for 10 min. The cells were washed with cold 1X PBS thrice, and harvested by scraping and centrifugation. The pellet was processed for ChIP-qPCR using SimpleChIP® Plus Enzymatic Chromatin IP Kit (CST, #9005). Chromatin was pulled down using H3K27Ac (Abcam, ab4729) antibody or H4K16Ac (Millipore, 07–329), and rabbit IgG antibody was used as negative control. The enrichment of modification was analyzed by qPCR. Primers used for qPCR are given Table 2.

### Western blot

Cell lysates were prepared using RIPA buffer (50 mmol/L Tris–HCl, pH 7.4; 150 mmol/L NaCl; 1% Triton X-100; 1% sodium deoxycholate) consisting of protease and phosphatase inhibitors. Equal protein concentration was loaded for all samples, separated on 12–18% polyacrylamide gel, and transferred onto PVDF membrane of pore size 0.45 µm. The transfer was carried out applying 300 mA constant current for 4 h at 4 °C. The membrane was blocked in 5% BSA in TBST for 1 h and incubated with primary antibody overnight at 4 °C. Further, membrane was washed 3 times with TBST and then incubated with secondary antibody for 1 h at RT. The blots were washed 3 times with TBST and developed using a chemiluminescent substrate (ClarityMax, Biorad). Antibodies used in study are provided in Table 3.

**Table 3:** Antibody used in the study

Protein	Company	Catalog number	Dilution	Purpose
H3	Sigma	H-0164	1:5000	WB
pAKT	Cell Signaling	43966	1:1000	WB
pH2AX	Millipore	05-636	1:5000	WB
H3K9ac	Millipore	04-1003	1:5000 1:100 1:100	WB IF IHC
H3K27ac	abcam	ab4729	1:5000 2 µg/10 µg chromatin	WB ChIP
H3K56ac	abcam	76307	1:3000	WB
H4K5ac	Millipore	06-759	1:5000	WB
H4K16ac	Millipore	07-329	1:8000 2 µg/10 µg chromatin 1:100	WB ChIP IF
H3K9Me3	abcam	8898	1:4000	WB
Pan-acetyl lysin	abcam	ab80178	1:1000	WB
Secondary anti-rabbit	Cell Signaling	7074	1:8000	WB
Secondary anti-mouse	Sigma	A-4416	1:5000	WB
Caspase 3	Cell signaling	9662	1:100	IHC
β-actin	Sigma	A-5316	1:10,000	WB

### Immunohistochemistry

The tissue sections of 4 μm thickness were mounted on glass slides coated with 0.1% poly-L-lysine. The sections were deparaffinized through a series of 4 xylene washes for 10 min, followed by xylene removal by absolute alcohol washes for 10 min. The tissue sections were rehydrated with 10 min of distilled water and peroxidase activity was quenched with 3% hydrogen peroxide. The antigen retrieval was carried out by heat treatment in a microwave for 5 min heating and 3 min cooling with 10 mM Tris buffer (pH 9.0). Then, 3 washes were given with 10 mM Tris (pH 7.5). Sections were blocked using horse serum for 30 min followed by incubation with primary antibody overnight in a humidified chamber at 4 °C. Further, the slides were washed using 10 mM Tris (pH7.5) buffer for 7 min 3 times each. Slides were then incubated with secondary antibody for 1 h at room temperature and then washed again. Then, HRP-conjugated-streptavidin was added to sections and incubated for 30 min at 30 °C. The development was done with diaminobenzidine staining for 4 min. The slides were washed under flowing tap water and counterstained with hematoxylin solution. The slides were then dehydrated and mounted with DPX. The slides were dried overnight at 37 °C. Pathologists carried out the immunohistochemical scoring based on percent intensity and area covered.

### Statistical analysis

Statistical calculations were performed using GraphPad Prism v9. Two-tailed Student's t test was performed for statistical analysis of quantitative data. P value < 0.05 was considered a statistically significant difference. Data with error bars represent average ± S.D. from biological triplicates.

## Results

### Development and characterization of cisplatin-resistant cancer cell line model

**Fig. 1:**
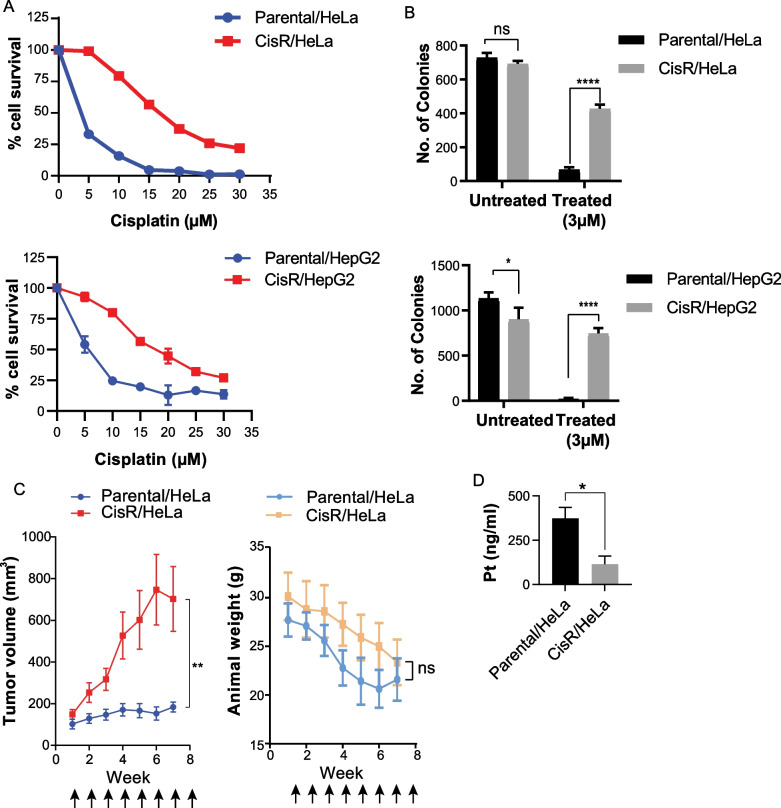
Establishment and characterization of cisplatin resistant cell lines. **A** Percent cell viability for the cell lines plotted as a line chart at different concentrations of the cisplatin. **B** Bar graph representing colonies obtained for cisplatin-resistant model. **C** Effect of cisplatin treatment on cisplatin resistant xenograft model. **D** Estimation of intracellular platinum concentration using TXRF method. Error bar represents mean ± S.D from 3 replicates. *p < 0.05, **p < 0.01, ***p < 0.001 and ****p < 0.0001

To study molecular changes in cisplatin-resistant cancer cell line, resistant model was developed using incremental dose exposure of cisplatin to parental cells. Cell viability assay was carried out to estimate IC_50_ by treating cells with increasing concentrations of cisplatin. The resistance index (RI) for the models was calculated using the standard formula. The RI value for the CisR/HeLa model was 4.5 and CisR/HepG2 was 3.1 (Fig. 1A). Further, the long-term survival ability of the model was assessed using clonogenic assay. A significantly higher number of colonies were observed in resistant cells than parental cells after drug treatment, suggesting that resistant cells have better long-term survival potential (Fig. 1B). A similar trend has been observed in in vivo HeLa cisplatin resistant xenograft tumors. After development of subcutaneous tumors with HeLa model system in NOD-SCID mice, animals were treated with 8 rounds of cisplatin. It was seen that CisR/HeLa cells showed significantly higher tumor volume over the course of treatment (Fig. 1C). The cisplatin treatment showed toxicity in animals as decrease in animal weight was observed. However, at the end of the experiment no animal had weight reduction more than 20% of initial. Thus, resistant cells also display better survival after drug treatment in xenograft experiments. We also observed significantly less drug accumulation inside CisR/HeLa cells (Fig. 1D). The low level of drug accumulation in resistant cells could be due to changes in import–export genes.

### Molecular alteration in cisplatin resistance displays enhanced PI3K-AKT signaling

**Fig. 2:**
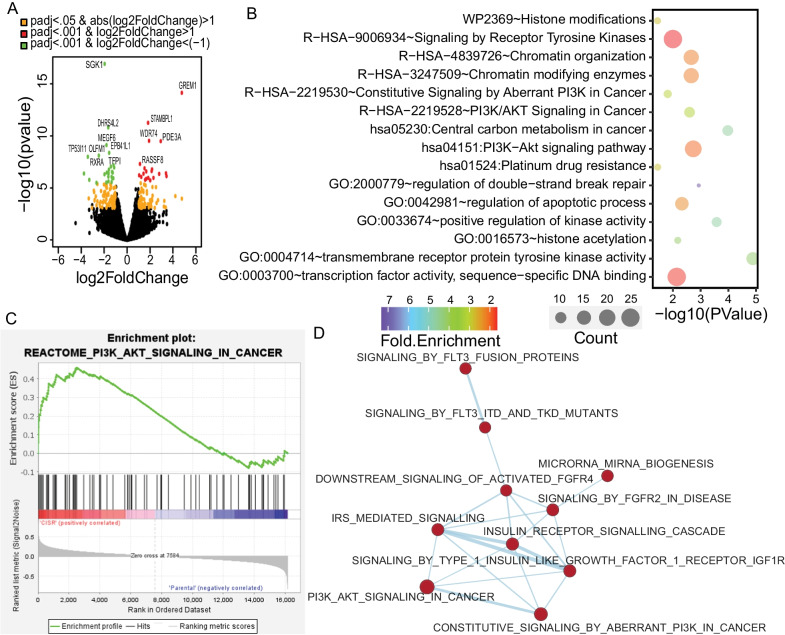
Differential gene expression and pathway analysis in cisplatin resistant model. **A** Volcano plot representing differentially expressed genes in parental vs. cisplatin-resistant cells. **B** Gene ontology and pathway analysis of upregulated in the cisplatin-resistant model. **C** Gene set enrichment analysis plot depicting enrichment of PI3K-AKT signaling in cisplatin resistant cells. **D** Network analysis of upregulated pathways in cisplatin resistance

To understand transcriptomic changes in cisplatin resistance, we analyzed RNA-seq data for parental vs cisplatin resistant cells from HeLa and HepG2 model. Cisplatin resistant cells showed 329 genes upregulated [log2FC > 1 & padj < 0.05] and 439 genes downregulated [log2FC < -1 & padj < 0.05] significantly. Differentially expressed genes were represented using volcano plot (Fig. 2A). Further, the transcriptome data was subjected to Gene Set Enrichment Analysis (GSEA) and pathway analysis. In case of cisplatin resistance, we found that resistant cells showed increased PI3K-AKT signaling pathway, central carbon metabolism, and epigenetic changes (Fig. 2B). GSEA data confirm significant enrichment of PI3K-AKT signaling pathway in resistant cells (Fig. 2C). Further, network analysis suggests that IGFR and FGFR mediated signaling pathways may play important role in cisplatin resistance along with altered pathways (Fig. 2D). We have carried out validation of transcriptome data for PI3K subunit genes, glucose metabolizing enzymes like HK2 &ENO1 and apoptosis inhibitor genes like BCLXL & XIAP using qPCR. All gene except BCLXL was found to be upregulated in both model systems (Additional file 1: Fig. S1A). Further, the activation of PI3K signaling was confirmed at protein level using western blot analysis of p-AKT levels in HeLa and HepG2 model system (Additional file 1: Fig. S1B). Similarly, to understand carbon metabolism, glucose uptake was checked in both models. It was found that cisplatin resistant cells have significantly higher glucose uptake (Additional file 1: Fig. S1C).

### Increase in histone acetylation is associated with cisplatin resistance

**Fig. 3:**
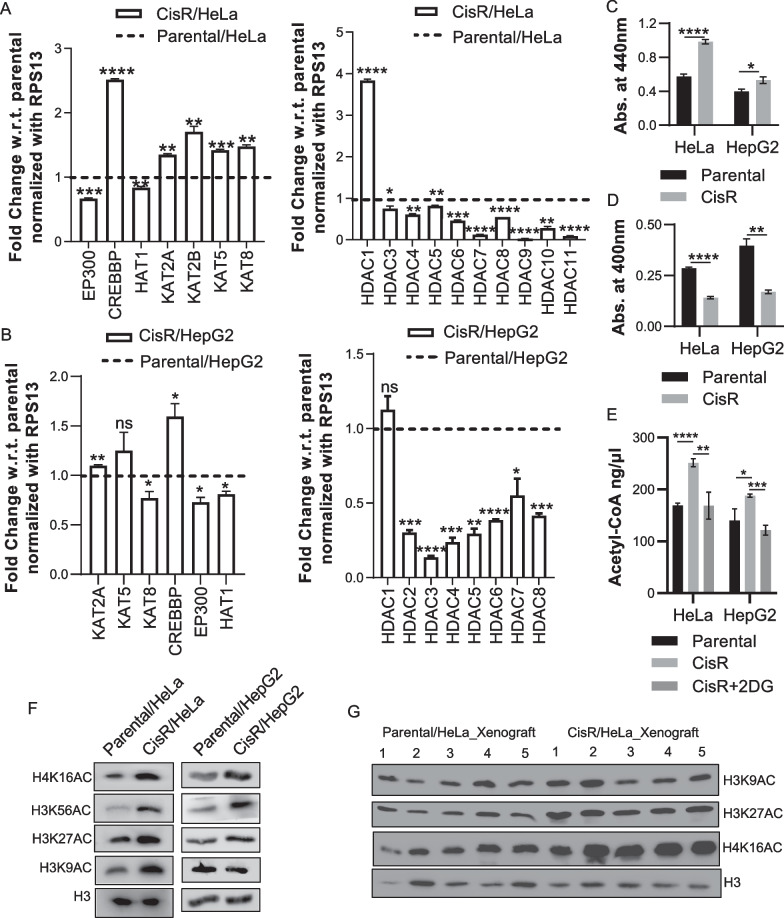
Alteration in histone modifying enzymes and their activity. **A**, **B** Bar graphs representing qPCR analysis for HAT & HDAC genes in HeLa and HepG2 cisplatin resistant model systems. **C** Estimation of HAT activity in cisplatin resistant cell lines. **D** Investigation of HDAC activity in resistant cells. **E** Assessment of acetyl-CoA levels in cisplatin-resistant cells. **F** Western blot analysis for histone lysine acetylation in in vitro cisplatin resistant models. **G** Western blot analysis for histone lysine acetylation in in vivo HeLa cisplatin resistant model. Error bar represents mean ± S.D from 3 replicates. *p < 0.05, **p < 0.01, ***p < 0.001 and ****p < 0.0001

HATs and HDACs were also found to altered in the transcriptome of both drug-resistant model. Based on qPCR validation, we confirmed that majority of HATs were upregulated, and HDACs were downregulated at the transcript level in cisplatin resistant model compared to parental (Fig. 3A, B). Further, we have performed enzyme activity assay for HATs and HDACs. It was observed that HAT activity was significantly high (Fig. 3C), and HDAC activity was significantly low in cisplatin resistant cells (Fig. 3D). Moreover, substrate levels for histone acetylation i.e., acetyl-CoA were also looked into parental vs resistant cells. It was seen that acetyl-CoA levels were high in resistant cells and upon 2-DG treatment acetyl-CoA levels drop significantly in resistant set (Fig. 3E). This suggests that cellular environment could be favorable for higher histone acetylation in cisplatin resistant cells.

In cisplatin resistance, we observed alterations in glucose uptake, imbalance in HATs/HDACs, and increased levels acetyl-CoA in resistant cells. Hence, we checked for histone acetylation levels in both resistant models as well as xenograft tissues of HeLa cisplatin resistant cells. We observed increase in histone acetylation levels in HeLa and HepG2 cisplatin resistant model (Fig. 3F). Also, it was observed that H3K27Ac and H4K16Ac were significantly increased in xenograft samples from HeLa cisplatin resistant model (Fig. 3G and Additional file 2: Fig. S2A). Since we have observed increase in PI3K signaling, it has been reported that phosphorylated AKT can activate ACYL allowing more synthesis of acetyl CoA from citrate. Also, it has reported that PI3K-AKT signaling can regulated the cellular growth. Hence, to understand correlation between histone acetylation and PI3K-AKT signaling, we treated HeLa resistant cells with PI3K inhibitor (Wortmanin) in combination with cisplatin for 24 h. We observed that histone acetylation levels decreased after Wortmanin treatment (Additional file 2: Fig. S2B). The data suggest that increased in increased in histone acetylation in resistant cells might be a consequence of active PI3K signaling. Moreover, it was noticed that a cell viability significantly decreases upon dual treatment indicating the importance of signaling pathway to tackle the toxic effects of cisplatin (Additional file 2: Fig. S2C).

### Role of histone acetylation in regulation of PI3K subunits and glycolysis genes

**Fig. 4:**
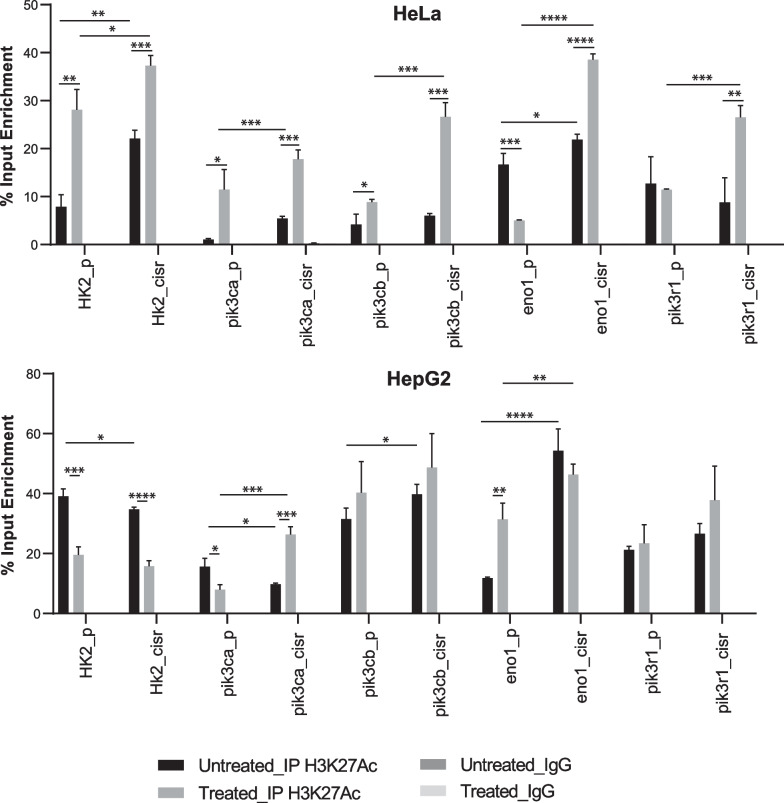
Enrichment of H3K27Ac in response to cisplatin treatment. Bar graph representing percent input enrichment of H3K27Ac on the promoter of depicted genes. The comparison carried out between parental & resistance w or w/o treated with cisplatin. Error bar represents mean ± S.D from 3 replicates. *p < 0.05, **p < 0.01, ***p < 0.001 and ****p < 0.0001

To understand the histone PTM mediated transcriptional activation of PI3K signaling or glycolysis in cisplatin resistance, we carried out ChIP with H3K27Ac and performed qPCR with promotor-specific primers for effectors of PI3K signaling pathway and glycolysis. We have also checked promotor enrichment upon treatment with cisplatin in parental and resistant cells in both the models. We observed that enrichment of H3K27Ac is more on the genes of PI3K subunits in resistant cells. Moreover, after cisplatin treatment, resistant cells display higher enrichment of the mark on PI3K genes. In case for glycolysis associated gene, HK2, HeLa model system shows higher enrichment of the mark in resistant cells compared to parental and cisplatin treatment increases enrichment in the enrichment, whereas contrasting results were observed in HepG2 model. The resistant cells possess low enrichment of the mark and upon cisplatin treatment the mark decreases on promotor of HK2 gene. ENO1 gene has higher enrichment in both cisplatin resistant models and even after drug treatment resistant cells carries higher enrichment on ENO1 gene promotor (Fig. 4). To confirm the gene activation on ENO1 gene, we perform ChIP with H4K16Ac and checked for its enrichment on ENO1 gene promotor. We observed that drug treatment alters the enrichment of H4K16Ac on ENO1, suggesting importance of the gene in response to cisplatin (Additional file 3: Fig. S3). Overall, we conclude that epigenetic regulation may play important role in regulating PI3K genes in resistant cells in response to cisplatin. Further, HK2 gene showed variation in two model systems for the gene activation. In HeLa model, we observe decrease in the enrichment of gene activation marks on ENO1 gene after drug treatment in parental cells. On the contrary, both resistant models possess higher enrichment of gene activation mark on ENO1 gene compared to parental cells irrespective of cisplatin treatment.

### Role of histone acetylation in survival of cisplatin resistant cells

**Fig. 5:**
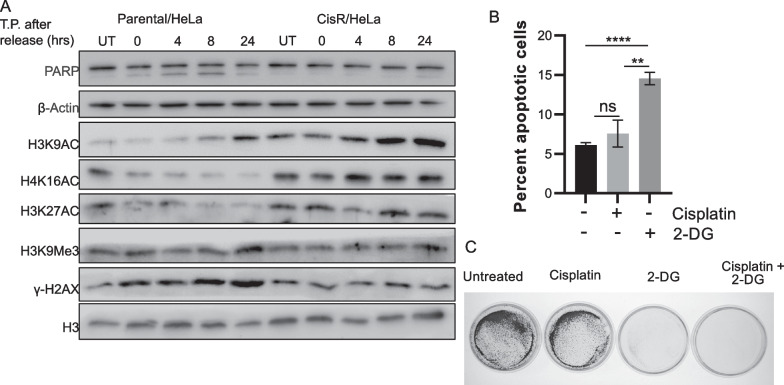
Role of histone acetylation in survival of cisplatin resistant cells. **A** Western blot analysis to check the effect of cisplatin in time-dependent manner on histone PTM levels. T.P. depicts Time point. **B** Percent apoptotic cells determined using Annexin-FITC/PI staining in CisR/HeLa cells. **C** Long-term survival assay upon 2DG treatment in CisR/HepG2 cells. Data with error bar represent mean ± S.D from 3 biological replicates *p < 0.05, **p < 0.01, ***p < 0.001 and ****p < 0.0001

Since we observe increase in histone acetylation levels in cisplatin resistant model, we tried to investigate the effect of drug treatment on histone acetylation of parental and resistant cells. Hence, we have treated HeLa parental and resistant cells with cisplatin for 24 h and then cells were grown in drug-free media for 24 h. The transitional time points 4,8, and 24 h after removal of the drug were studied for the induction of apoptosis using PARP cleavage, DNA damage with γH2AX levels, and histone acetylation changes. Western blot data showed a time-dependent increase in PARP cleavage in the parental cell after drug treatment, indicating the induction of apoptosis. Also, an increase in γH2AX suggests that more DNA damage has occurred in parental cells compared to resistant cells. On the other hand, PTM profiling in the showed that H3K9Ac, H3K27Ac, and H4K16Ac were unchanged even in drug treatment in cisplatin-resistant cells, whereas parental cells show a decrease in these marks over the period (Fig. 5A). Further, H3K9Me3 levels remain unchanged in both the experimental group, which depicts that acetylation might play a role in the maintenance of chemoresistance.

To understand the importance of histone acetylation, we decided to limit the substrate concentration to alter the acetylation levels using 2-Dexoy-Glucose. Treatment of 25 mM 2DG alone or in combination with 3 μM cisplatin for 24 h was found to be sufficient to induce cell death in cisplatin-resistant HeLa cells (Fig. 5B). Further, a clonogenic assay was carried out in a HepG2 cisplatin-resistant model with the dual treatment of 3 μM cisplatin and 25 mM 2DG. It was observed that glucose limitation decreases the long-term survival of resistant cells (Fig. 5C). Overall, in vitro data indicate that glycolysis process, PI3K signaling and histone acetylation have critical role in survival of cervical and liver cancer cisplatin-resistant cells.

### Treatment with 2DG reduces growth of cisplatin resistant tumors due to induction of cell death

**Fig. 6:**
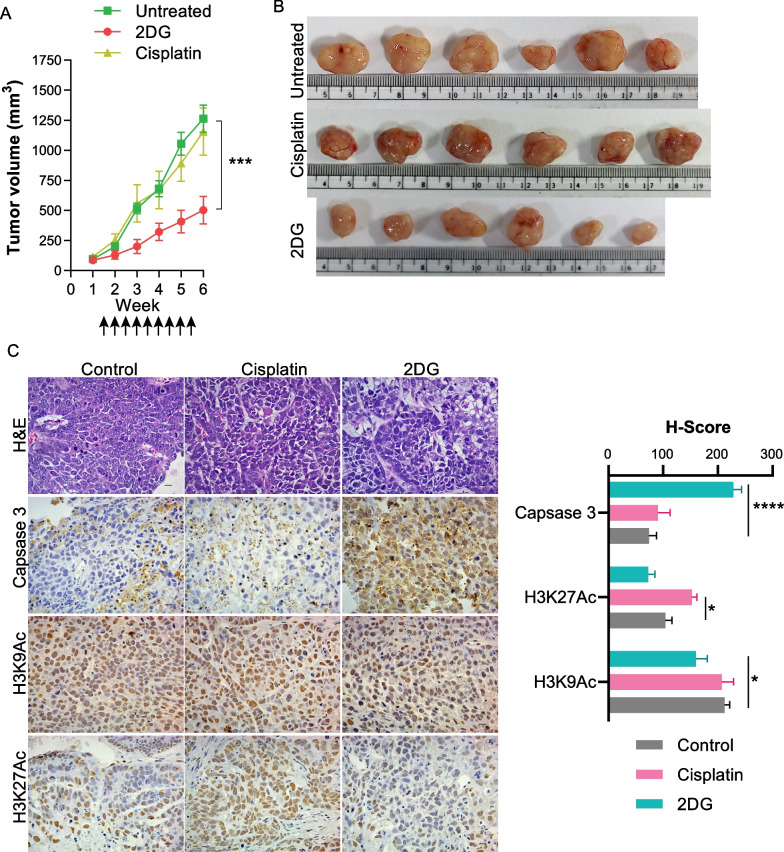
Effect of 2DG treatment on tumorigenic potential of cisplatin resistant cells.** A** Estimation of tumor progression of CisR/HeLa cells after Cisplatin or 2DG treatment. ↑ represents 1 round of dose.** B** Representative image for tumor size after 8 weeks of treatment in different groups.** C** Effect of 2DG on histone PTMs and Caspase3 in cisplatin resistant xenograft tissues. Bar graph representing the H-Score desired antigens in the experimental setup. The scale bar denotes 10 μm. The bar graph represents H-Score for mean ± S.E.M. from 4–5 biological replicates. *p < 0.05, and ****p < 0.0001

To check the in vivo efficacy of 2DG, we injected cisplatin-resistant HeLa cells subcutaneously in NOD-SCID mice and treated animals with cisplatin or 2DG after tumor development. It was observed that tumor progression was reduced, and tumor volume was significantly less at the end of the 8th week in 2DG-treated animals compared to the untreated group (Fig. 6A, B). Cisplatin-treated animals showed similar tumor progression and tumor volume compared to untreated. The histopathological analysis showed necrosis in tumor sections from all three groups in H&E-stained sections. The possible reason for necrosis in the control and the cisplatin-treated group is higher tumor size leading to necrotic tumor core. However, the 2DG-treated group showed higher percent necrosis normalized to tumor volume.

Moreover, IHC with caspase 3, H3K9Ac, and H3K27Ac was carried out to understand the effect of 2DG on histone post-translational modifications and apoptosis markers, and results were displayed using H-Score (Fig. 6C). The data suggest that animals treated with 2DG have significantly high cytoplasmic caspase 3 levels suggesting induction of cell death due to 2DG treatment (Fig. 6C). Histone acetylation H3K27Ac and H3K9Ac showed low nuclear intensity in 2DG-treated sets indicating the effect of 2DG on the decrease in acetylation levels. Moreover, in the cisplatin-treated group, these marks showed an increasing trend correlating with in vitro data. In vitro and in vivo pharmacological perturbation of carbon metabolism by 2DG in resistant cells revealed that direct metabolic alteration can be a potent driving force for changes in histone acetylation patterns, leading to cell death.

## Discussion

Despite emerging new drugs for cancer treatment, limited options are available for advanced or recurring tumors. By understanding the mechanisms of resistance, recurrent tumors can be targeted with an alternative treatments or combination of treatments. While various explanations for drug resistance have been put forth, the focus of this study is to investigate the molecular landscape of deregulated pathways, cross talk with histone acetylation and identify potential targets in cisplatin resistance. According to McDermott et al., drug resistance can vary among different types of tissue, with a clinically relevant resistance index ranging from 3 to 8 [14].

In this study, we have developed the cisplatin resistant cell lines for HeLa & HepG2. Further, transcriptome profile suggests that PI3K-AKT signaling has upregulated in cisplatin resistant cells. The PI3K-AKT signaling can regulate multiple pathways downstream of its effector module AKT [15]. Moreover, extracellular growth factors from serum can activate the pathway by ligand-receptor interaction followed by converging signaling cascade at phosphorylated AKT. Increased AKT phosphorylation has been linked to improved cell viability, migration, and resistance to apoptosis in breast cancer and glioblastoma cells, potentially causing chemoresistance [16, 17]. The inhibition of AKT/mTOR has been shown to increase CDDP-induced apoptosis by increasing Bax and releasing cytC in resistant cells [18, 19]. Activation AKT contributes to CDDP resistance through suppressing P53 [20]. PI3K/AKT-mediated translocation of β-catenin to the nucleus leads to upregulation of genes linked to drug transporters and multi-drug resistance [21, 22]. However, success in for PI3K pathway inhibitors has faced a major challenge. Pan-PI3K inhibitors have grade 3–4 toxicities consisting of rash, fatigue, hyperglycemia, and diarrhea [23]. Cancer cells have been shown to adapt the inhibition of the PI3K pathway through non-genetic mechanisms like feedback upregulation of compensatory mechanisms. Additionally, resistance to PI3K inhibitors has been attributed to mutations in genes like KRAS, PIK3CA, PIK3CB, and the amplification of CDK4/6, c-MYC, FGFR1.

PI3K-AKT signaling has been shown to alter glucose availability and production of cytosolic acetyl-CoA intern regulating histone acetylation levels in cancer cells [24]. Cells that undergo proliferation and have elevated levels of glucose uptake, do not entirely oxidize all of their mitochondrial citrate. Instead, some of the citrate is transported to the cytosol, where it can be converted to cytosolic acetyl-CoA through the action of ATP-citrate lyase (ACL). The activity of ATP-citrate lyase is regulated by p-Akt [25, 26]. Further, it has been shown that AKT-mediated phosphorylation of p300/CBP enhances the acetylation potential of the HATs [27]. Our observations have shown an increase in HAT activity, glucose uptake, intracellular acetyl-CoA levels, and a decrease in HDAC activity, leading to an imbalanced relationship between HAT and HDAC and a subsequent increase in histone acetylation. Class II and III HDACs have shown to modulate activity, stability, localization and interactions of proteins involved in gene transcription, DNA damage repair, cell division, signal transduction, protein folding, autophagy and metabolism [28]. However, in cisplatin resistance it needs further investigation. It has been shown that BRCA2 N372H modulates HAT activity, conferring paclitaxel resistance in breast cancer [29]. Moth et al. found a higher enrichment of H3K9Ac on the MDR1 gene in the drug-resistant breast cancer cell line [30]. Further, BRD4 enrichment was observed on the RUNX2 gene, which drives essential genes from drug resistance in osteosarcoma cells [31]. Our ChIP data suggest that higher abundance of histone acetylation is one of responsible factor for enhanced PI3K-AKT signaling and glycolysis. The increase in PI3K promoter enrichment after cisplatin treatment highlights the importance of the PI3K-AKT signaling in drug resistance. Histone acetylation may also regulate other critical genes involved in resistance.

In resistant cells, histone acetylation persists after cisplatin treatment. To stop tumor cell growth, it is crucial to inhibit histone acetylation, but currently, there are no histone acetyltransferase inhibitors in clinical or preclinical trials. Another way to control histone acetylation is by reducing the acetyl-CoA levels, which is produced from glycolysis. There is growing evidence that abnormal glycolysis contributes significantly to drug resistant cancer cells and that dysfunctional glycolysis is frequently linked to treatment failure. To lower acetyl-CoA amount, the glycolytic pathway is a key target. Certain glycolytic intermediates can inhibit the pathway through feedback loop mechanisms if present in excess. 2-Deoxy-glucose (2-DG) is a glucose analog that has been found to be effective in limiting the growth of tumor cells. It works by blocking the glycolytic pathway, which is a key source of energy for cancer cells. By blocking this pathway, 2-DG reduces the production of acetyl-CoA, which is needed for histone acetylation. The reduction in histone acetylation, in turn, restricts the growth of tumor cells. Xu et al. have shown that 2-DG enhances the apoptosis effect of TRAIL by suppressing the c-Jun N-terminal kinase (JNK)-protected autophagy processes [32]. Additionally, 2-DG has been proven to raise the generation of ROS and block the pentose phosphate shunt, which leads to cell death [33]. In a pancreatic tumor experiment, 2-DG was shown to make cells more susceptible to 5-FU, demonstrating its specific impact on cells [34]. These findings support our data for reduction in tumor growth of in cisplatin resistant cells. Interestingly, we also observed that 2DG can affect non-resistant cancer cells which possibly due to Warburg effect (data not shown).

**Fig. 7:**
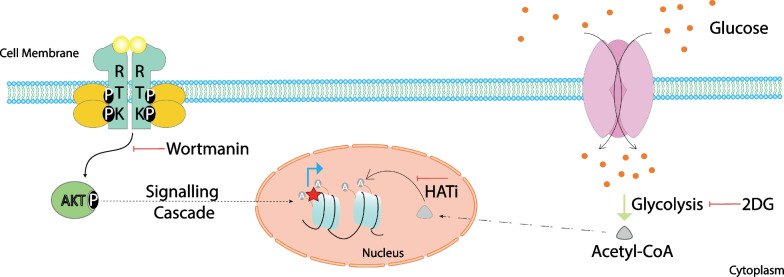
Schematic representation highlighting the molecular alterations in cisplatin resistant model. Cisplatin-resistant cells display more activation of PI3K signaling, Acetyl-CoA production and histone hyperacetylation. The occupancy of histone acetylation marks on the promoter of genes related to PI3K subunits and glycolysis in resistant cells indicates their importance. Inhibiting glycolysis using 2-DG or PI3K signaling may imporove therapeutic outcome in terms of reduces tumor growth

In conclusion, histone acetylation plays vital role in governing cisplatin resistance in cervical and liver cancer cells. Occurrence of acetylation on promoters of key genes regulating PI3K signaling and glycolysis pathway highlights their  importance in cisplatin resistance (Fig. 7). Inhibiting glycolysis using 2-DG was found to reduce tumor growth in cisplatin-resistant cells. However, combating cisplatin-resistance by targeting metabolism and histone acetylation in a patient sample will be a step forward. Moreover, in the advanced or chemoresistant tumors with limited treatment modalities, the low-dose chemotherapy targeting defined metabolic reprogramming and closely associated alterations can partially shrink tumors to extend survival and improve quality of life.

### Supplementary Information

**Additional file1: Fig. 1**. Validation of transcriptome data in cisplatin resistant mode systems. A. Heatmap representing Log_2_(Fold Change) in cisplatin-resistant cells with respect to parental cells for desired genes. B. Western blot analysis for p-AKT levels in the cisplatin-resistant models. Numbers writtern blot represents band intensity of protein normalized with β-actin. C. Graph indicating percent glucose uptake normalized with media control in HeLa & HepG2 model. Data with error bar represent mean ± S.D from 3 biological replicates. p < 0.05=*, p < 0.01=**.

**Additional file 2: Fig. 2**. Correlation between histone acetylation and PI3K-AKT signaling. A. Densitometric analysis of histone PTMs from in vivo samples normalized with H3 levels. B. Western blot analysis for histone PTMs in resonse to PI3K-AKT pathway inhibition CisR/HeLa model. C. Percent live cells after combinatorial drug treatment in cisplatin resistant HeLa cells. Data with error bar represent mean ± S.D from atleast 3 biological replicates. p < 0.05=*, p < 0.01=**, p < 0.001=*** and p < 0.0001=****.

**Additional file 3: Fig. 3**. Enrichment of H4K16Ac on ENO1 gene promoter in cisplatin resistance. Data with error bar represent mean ± S.D from 3 biological replicates. p < 0.05=*, p < 0.01=**, p < 0.001=*** and p < 0.0001=****.

## Data Availability

Any supporting data will be made available with the first and corresponding author and can be shared upon request. RNA-seq data used in study are available under NCBI bioproject ID PRJNA1017179 and PRJNA874573.

## Abbreviations

CSCs: Cancer stem cells
DDR: DNA damage repair
HAT: Histone acetyltransferase
HDAC: Histone deacetylases
