# Supplementary figures and images for: Histone acetylation: a key determinant of acquired cisplatin resistance in cancer
Source: Clin Epigenetics. 2024 Jan 3;16:8. doi: 10.1186/s13148-023-01615-5 (PMC10765630; doi:10.1186/s13148-023-01615-5)

A

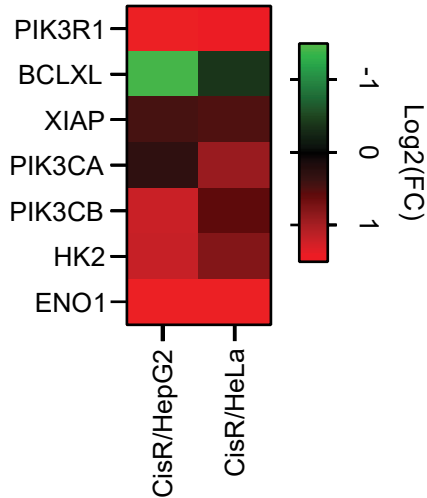

B

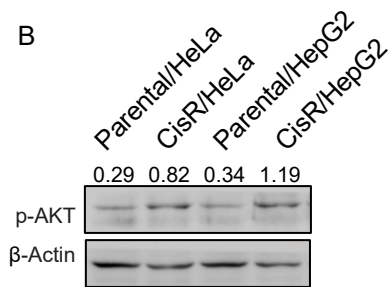

C

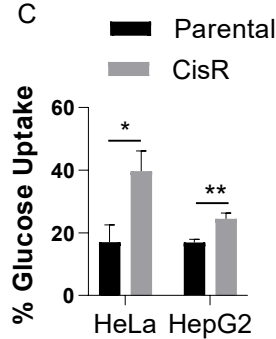

Supplement: Supplementary file 1 — Additional file1: Fig. 1. Validation of transcriptome data in cisplatin resistant mode systems. A. Heatmap representing Log2(Fold Change) in cisplatin-resistant cells with respect to parental cells for desired genes. B. Western blot analysis for p-AKT levels in the cisplatin-resistant models. Numbers writtern blot represents band intensity of protein normalized with β-actin. C. Graph indicating percent glucose uptake normalized with media control in HeLa & HepG2 model. Data with error bar represent mean ± S.D from 3 biological replicates. p < 0.05=*, p < 0.01=**. [file 13148_2023_1615_MOESM1_ESM.pdf]

C

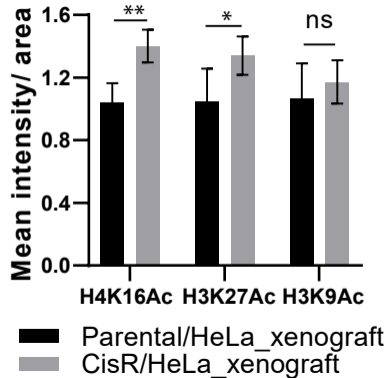

D

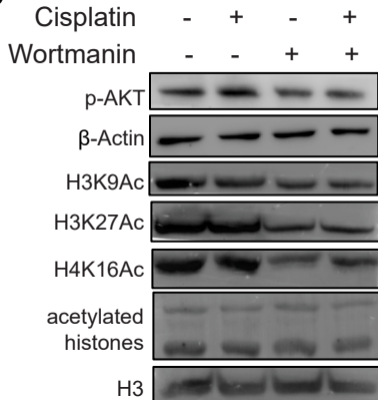

E

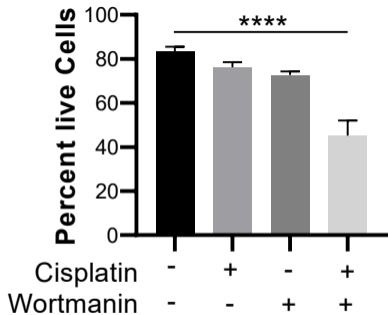

Supplement: Supplementary file 2 — Additional file 2: Fig. 2. Correlation between histone acetylation and PI3K-AKT signaling. A. Densitometric analysis of histone PTMs from in vivo samples normalized with H3 levels. B. Western blot analysis for histone PTMs in resonse to PI3K-AKT pathway inhibition CisR/HeLa model. C. Percent live cells after combinatorial drug treatment in cisplatin resistant HeLa cells. Data with error bar represent mean ± S.D from atleast 3 biological replicates. p < 0.05=*, p < 0.01=**, p < 0.001=*** and p < 0.0001=****. [file 13148_2023_1615_MOESM2_ESM.pdf]

## HeLa

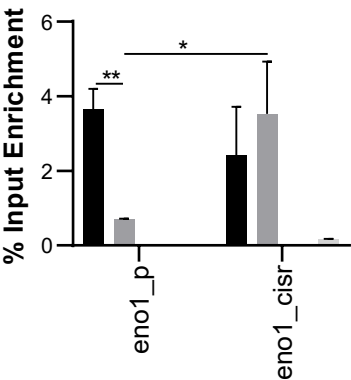

Untreated\_IP H4K16Ac  
 Treated\_IP H4K16Ac

## HepG2

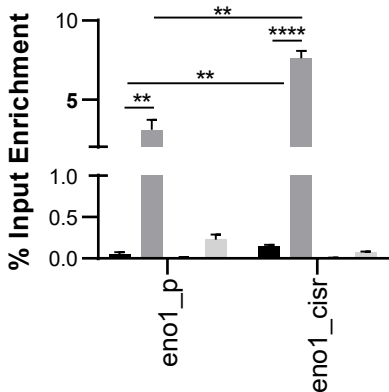

Untreated\_IgG  
 Treated\_IgG

Supplement: Supplementary file 3 — Additional file 3: Fig. 3. Enrichment of H4K16Ac on ENO1 gene promoter in cisplatin resistance. Data with error bar represent mean ± S.D from 3 biological replicates. p < 0.05=*, p < 0.01=**, p < 0.001=*** and p < 0.0001=****. [file 13148_2023_1615_MOESM3_ESM.pdf]
